# Repeat expansion disorders

**DOI:** 10.1136/pn-2023-003938

**Published:** 2024-09-30

**Authors:** Zhongbo Chen, Huw R Morris, James Polke, Nicholas W Wood, Sonia Gandhi, Mina Ryten, Henry Houlden, Arianna Tucci

**Affiliations:** 1 Department of Clinical and Movement Neuroscience, University College London Queen Square Institute of Neurology, London, UK; 2 The Francis Crick Institute, London, UK; 3 The Neurogenetics Laboratory, National Hospital for Neurology and Neurosurgery, University College London Hospitals NHS Foundation Trust, London, UK; 4 UK Dementia Research Institute at University of Cambridge, Cambridge, UK; 5 Department of Neuromuscular Disease, University College London Queen Square Institute of Neurology, London, UK; 6 William Harvey Institute, Queen Mary University of London, London, UK

**Keywords:** GENETICS, CEREBELLAR ATAXIA, MOLECULAR BIOLOGY, NEUROGENETICS

## Abstract

An increasing number of repeat expansion disorders have been found to cause both rare and common neurological disease. This is exemplified in recent discoveries of novel repeat expansions underlying a significant proportion of several late-onset neurodegenerative disorders, such as CANVAS (cerebellar ataxia, neuropathy and vestibular areflexia syndrome) and spinocerebellar ataxia type 27B. Most of the 60 described repeat expansion disorders to date are associated with neurological disease, providing substantial challenges for diagnosis, but also opportunities for management in a clinical neurology setting. Commonalities in clinical presentation, overarching diagnostic features and similarities in the approach to genetic testing justify considering these disorders collectively based on their unifying causative mechanism. In this review, we discuss the characteristics and diagnostic challenges of repeat expansion disorders for the neurologist and provide examples to highlight their clinical heterogeneity. With the ready availability of clinical-grade whole-genome sequencing for molecular diagnosis, we discuss the current approaches to testing for repeat expansion disorders and application in clinical practice.

## The burden of neurogenetic disorders

 There is a significant burden of genetic disorders in neurology, with at least half of the 8000 or so described rare genetic diseases having some neurological manifestation.^1 2^ Even though each of these rare diseases affects fewer than 1 in 2000 individuals, collectively they are common, with the estimated cumulative lifetime risk of developing such a disorder being 1 in 17 people.^3 4^

There has been steady progress in the diagnosis of neurogenetic diseases over the last two decades, driven by advances in the approach to genetic testing.^2^ The shift away from single-gene testing conducted by specialist services to the mainstreaming of genomic medicine to all specialties has been accompanied by the widespread availability of clinical-grade whole-genome sequencing (WGS)^5^ in the National Health Service (NHS) in England (https://www.england.nhs.uk/genomics/nhs-genomic-med-service/). Despite this, the diagnostic yield remains relatively low with pitfalls of WGS discussed in a recent review.^6^ Up to 80% of patients with a genetic neurological presentation go undiagnosed even in the 100 000 Genomes Project, an initiative to assess the utility of WGS in NHS patients with rare disease and cancer.^7^

One such prevailing bottleneck to molecular diagnosis is the limitation in conventional techniques to detect structural variation, defined as regions of genomic variation greater than 50 base pairs in length compared with the reference genome.^8 9^ This is a significant concern as structural variants account for up to 20% of all rare genomic variation^10^ and are particularly important in neurological disease.

## What are repeat expansion disorders?

**Figure 1:**
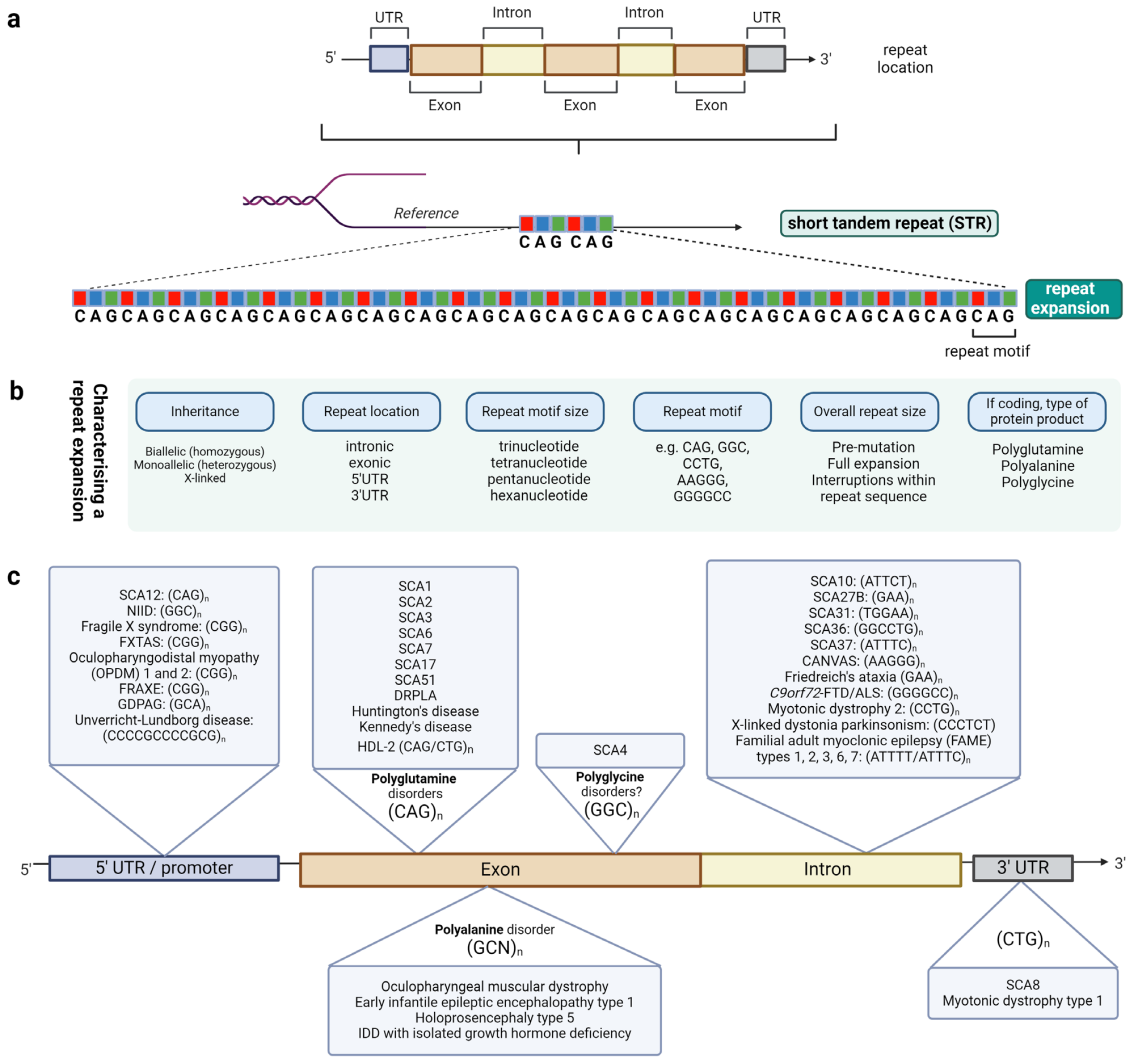
Repeat expansion disorders arise from short tandem repeats (STRs) that reside naturally within the genome (**a**). STRs can arise from any genic position and comprise repeat sequences of up to six base pairs in tandem. When expanded above a certain pathogenic threshold, they are associated with a repeat expansion disorder.While STRs can be intergenic, no pathogenic expansions of intergenic STRs have been characterised to date. UTR refers to untranslated regions of the gene. (**b**) Features of a repeat expansion that influence pathogenesis and are used to characterise a repeat expansion disorder. (**c**) Non-exhaustive list of repeat expansion disorders associated with neurological disease represented by their intragenic location. The repeat motif is listed after the disease in parentheses or collectively, within the triangles. n represents the number of repeat units. N in GCN represents any A/C/G/T nucleotide. The exonic repeat expansions are further partitioned by the probable translated product of the repeat-containing DNA into polypeptides, namely polyglutamine, polyglycine and polyalanine disorders. Unverricht-Lundborg disease is listed but is a dodecamer repeat and thus not strictly an expansion of an STR (each repeat unit is up to six base pairs). ALS, amyotrophic lateral sclerosis; CANVAS, cerebellar ataxia, neuropathy, vestibular areflexia syndrome; DRPLA, dentatorubral-pallidoluysian atrophy; FAME, familial adult myoclonic epilepsy; FTD, frontotemporal dementia; FXTAS, fragile X-associated tremor/ataxia syndrome; FRAXE, intellectual developmental disorder, X-linked; GDPAG, global developmental delay, progressive ataxia, and elevated glutamine; HDL-2, Huntington disease-like 2; IDD, intellectual development disorder; NIID, neuronal intranuclear inclusion disease; OPDM, oculopharygodistal myopathy; SCA, spinocerebellar ataxia. Created with BioRender.com.

Expansions of short tandem repeats (STRs) are one such category of structural variation. An STR comprises repetitive DNA sequences of up to six base pairs in tandem^11 12^ (figure 1a). There are over a million such naturally occurring STRs in each individual genome with high variability in repeat size between different people.^13^ As such, STR genotyping has been traditionally used as a marker of forensic DNA profiling.^14^ STRs have been implicated in controlling gene expression^12^ and driving human-specific evolution.^15 16^

When a particular STR is repeated multiple times beyond a pathogenic threshold (figure 1a), the so-called *repeat expansion*, its presence can be causative for disease, termed *a repeat expansion disorder*. It should be noted that the pathogenic threshold for repeat size varies between different disorders at different genetic loci. Box 1 summarises the inter-related ways in which repeat expansions cause disease.^17^ Commonalities in the clinical presentation, overarching diagnostic features, similarities in the approach to genetic testing and targeted therapeutics of repeat expansions justify considering these conditions collectively based on their unifying molecular cause.

Box 1 **How repeat expansions can cause disease.**

The repetitive DNA sequence can cause disease in several inter-related ways. Multiple mechanisms can coexist within the same disease entity.^17^

- The abnormal repeat is translated into a toxic polypeptide, such as in CAG repeat expansions leading to polyglutamine disorders including spinocerebellar ataxia (SCA) types 1, 2, 3, 6 and 7.
- The abnormal repeat expansion causes the gene to be switched off (transcriptional silencing) leading to loss of function of that gene, such as in fragile X syndrome.
- The repeat-containing RNA may block important proteins from functioning such as RNA-binding proteins that have important roles in transcription and splicing such as in myotonic dystrophy types 1 and 2.
- The abnormal repeat initiates translation to form toxic peptides non-canonically, termed repeat-associated non-AUG translation, a mechanism thought to contribute to the pathogenicity of *C9orf72-*associated frontotemporal dementia/amyotrophic lateral sclerosis, SCA8 and other repeat expansion disorders.

## Why are repeat expansion disorders important in neurology?

It is not known why repeat expansion disorders are particularly enriched among genetic causes of both rare and complex neurological disease. Since the first repeat expansion in an exonic CAG repeat in the androgen receptor (*AR*) gene was characterised as causative for spinal and bulbar muscular atrophy (Kennedy’s disease) in 1991,^18^ most of the repertoire (current count at around 60 or so) of repeat expansion disorders described to date are associated with neurological or neurodevelopmental disease.^19^,^21^ There are only a handful of non-neurological syndromes associated with repeat expansions including Fuchs endothelial corneal dystrophy,^22^ synpolydactyly type 1^23^ and hand-foot-genital syndrome.^24^

A pathogenic repeat expansion can sometimes account for a significant proportion of a neurodegenerative disease as exemplified through the *C9orf72* hexanucleotide repeat expansion, which is the the most common genetic cause of amyotrophic lateral sclerosis (ALS) and frontotemporal dementia (FTD) and occurs in both apparently sporadic and familial patients.^19 20^ Furthermore, recently discovered *RFC1* expansions associated with cerebellar ataxia, neuropathy and vestibular areflexia syndrome (CANVAS)^25^ and GAA repeat expansion in *FGF14* associated with spinocerebellar ataxia (SCA) 27B^26 27^ have been found to explain a high proportion of previously undiagnosed individuals with late-onset ataxia. This highlights the necessity of considering repeat expansions as a potential cause of neurological disease among those without a molecular diagnosis.

Repeat expansion disorders manifest across a host of neurological phenotypes reflecting involvement of different regions from the central to the peripheral nervous system, meaning that most neurologists will regularly encounter such disorders. The range of neurological presentations is broad and includes, but is not limited to: epilepsy (eg, familial adult myoclonic epilepsies (FAME)); ataxia (eg, SCAs, CANVAS and Friedreich’s ataxia); cognitive impairment (eg, *C9orf72-*associated frontotemporal dementia and ALS); movement disorders (eg, Huntington’s disease, X-linked dystonia-parkinsonism); learning difficulty (eg, fragile X syndrome) and neuromuscular disorders (eg, myotonic dystrophy types 1 and 2, oculopharyngeal muscular dystrophy (OPMD)). In many repeat expansion disorders, multiple phenotypes coexist under one disease entity reflecting the underlying multisystemic involvement and clinical heterogeneity.

From epidemiological studies, repeat expansion disorders affect 1 in 3000 people worldwide, with population differences across different diseases.^28^ However, clinical heterogeneity across the disorders likely leads to an underestimation of the underlying prevalence. Leveraging large-scale population sequencing data, a recent study found that the overall disease allele frequency of repeat expansion disorders is more common than previously thought, affecting 1 in 283 individuals.^28^

In summary, repeat expansion disorders manifest across a wide range of neurological presentations and may bypass the bottleneck to diagnosis. Knowledge of their characteristic clinical features and how to request testing for such disorders is of increasing significance in clinical practice.

## How do we characterise repeat expansions molecularly?

Repeat expansion disorders can be characterised across different domains (figure 1b). It is important to be aware of factors that influence their pathogenicity. These include: (1) the overall size of a repeat; (2) its location within a gene; (3) the motif of the repeat unit; (4) how it is inherited including biallelic (recessive) or monoallelic (dominant) inheritance; (5) whether the repeat-DNA is transcribed into RNA and translated into protein; (6) the stability of the repeat size across different generations (meiotic instability) and across cell types within an individual (somatic instability). Diagnostic reports typically define the first four points only.

A STR expansion can be characterised by its location within a gene, that is, broadly, whether it is in the 5’ or 3’ untranslated regions (UTR), intron or exon. This has implications for the overall repeat size as pathogenic repeats in non-coding genic regions tend to be larger than exonic repeats, such as a pathogenic threshold of more than 200 CGG repeats in the 5’UTR of *FMR1* associated with fragile X syndrome^29^ compared with a threshold of 36–39 exonic CAG repeats in *ATXN1* associated with SCA1.^30^ Exonic repeats are typically protein coding and can be characterised by their protein product. There are currently three main types of protein-coding repeats secondary to the different underlying repeat motifs which code for different amino acids. These are: (1) the well-known polyglutamine repeats from exonic CAG repeat expansion; (2) polyalanine repeat expansion disorders associated with congenital malformation syndromes with the exception of the *PABPN1* GC*N* repeat expansion associated with OPMD (where *N* could be an A/T/C/G nucleotide)^31^ and (3) the new disease entity of polyglycine disorders identified after the discovery of an exonic GGC repeat expansion in *ZFHX3* associated with SCA4.^32^,^35^

The repeat motif can determine whether an STR expansion is pathogenic. For example, in CANVAS, the most common repeat expansion is of the AAGGG unit ranging from 400 to more than 2000 repeats while AAAAG, AAAGGG and AAGAG expansions appear to be benign.^25 36 37^ There are also population differences in the pathogenic repeat motif such as a biallelic ACAGG repeat expansion associated with CANVAS in individuals of Japanese ancestry.^38^ Of note, several repeat expansion disorders discovered in recent years are insertional expansions where the pathogenic repeat motif is not found in the reference genome or in unaffected individuals (table 1).^39^ This is seen across the FAME disorders where the pathogenic pentanucleotide expansion may have variable motifs.^40^ In X-linked dystonia-parkinsonism, a disorder characterised by progressive dystonia and extrapyramidal syndrome, there is a SINE-VNTR-Alu (SVA) retrotransposon insertion in the intron of *TAF1* where a 5’ polymorphic hexanucleotide CCCTCT repeat is located. The length of this repeat insertion is inversely correlated with age of onset.^19^

**Table 1:** Repeat expansion disorders associated with neurological disease

Disease	Gene	Repeat motif	Normal range	Pathogenic repeat number	Location	Inheritance	Phenotype
Spinocerebellar ataxia 1	*ATXN1*	CAG	6–39	>39	Exon	AD	Ataxia, neuropathy, pyramidal signs
Spinocerebellar ataxia 2	*ATXN2*	CAG	15–29	>34	Exon	AD	Ataxia, neuropathy, slow saccades, cognitive impairment, Cuban founder effect
Spinocerebellar ataxia 3	*ATXN3*	CAG	13–36	55–84	Exon	AD	Ataxia, pyramidal and extrapyramidal signs, large, Portuguese founder effect
Spinocerebellar ataxia 4	*ZFHX3*	GGC	<21–30	>48	Exon	AD	Ataxia, sensory axonal neuropathy, Swedish founder effect
Spinocerebellar ataxia 6	*CACNA1A*	CAG	4–16	21–30	Exon	AD	Slowly progressive ‘pure’ ataxia
Spinocerebellar ataxia 7	*ATXN7*	CAG	3–35	34 to >300	Exon	AD	Ataxia, visual loss with retinopathy
Spinocerebellar ataxia 8	*ATXN8/ATXN8OS*	CAG	6–37	~107–250	3'UTR	AD	Ataxia, slowly progressive, sensory neuropathy
Spinocerebellar ataxia 10	*ATXN10*	ATTCT	10–29	280–4500	Intron	AD	Ataxia, seizures
Spinocerebellar ataxia 12	*PPP2R2B*	CAG	<66	>66	5'UTR	AD	Ataxia, action tremor, hyper-reflexia, extrapyramidal features
Spinocerebellar ataxia 17	*TBP*	CAG	25–44	45–66	Exon	AD	Ataxia, chorea, dystonia, myoclonus, epilepsy
Spinocerebellar ataxia 27B	*FGF14*	GAA	6–249	>300	Intron	AD	Late-onset ataxia, may have episodic onset, downbeat nystagmus, vertigo, neuropathy
Spinocerebellar ataxia 31	*BEAN1*	TGGAA	NA (as insertion)	>500	Intron	AD	Late-onset ataxia, Japanese founder effect
Spinocerebellar ataxia 36	*NOP56*	GGCCTG	3–8	1500–2500	intron	AD	Ataxia, eye movement abnormalities, tongue fasciculations, upper motor neuron signs
Spinocerebellar ataxia 37	*DAB1*	ATTTC	NA (as insertion)	Insertion	5'UTR	AD	Slowly progressive gait and limb ataxia, eye movement abnormalities, dysphagia
Spinocerebellar ataxia 51	*THAP11*	CAG	19–39	45–100	Exon	AD	Late-onset ataxia, described in 2 Chinese families
Dentatorubral-pallidoluysian atrophy	*ATN1*	CAG	7–25	>49	Exon	AD	Ataxia, chorea, dementia, myoclonus, seizure, anticipation, common in Japan
Cerebellar ataxia, neuropathy and vestibular areflexia syndrome	*RFC1*	AAGGG	AAAAG:11–200, AAAGG 40–1000	AAGGG 400- >2000 Different motifs	Intron	AR	Late-onset ataxia, sensory neuropathy, vestibular areflexia syndrome
Friedreich’s ataxia	*FXN*	GAA	7–22	>66	Intron	AR	Ataxia, scoliosis, bladder dysfunction, absent lower-limb reflexes, and loss of position and vibration sense, cardiomyopathy, diabetes (later onset in 25%)
Fragile X-associated tremor/ataxia syndrome	*FMR1*	CGG	6–52	55–200	5'UTR	X-linked	Late-onset ataxia, intention tremor, followed by cognitive impairment
Fragile X syndrome	*FMR1*	CGG	6–52	>200	5'UTR	X-linked	Developmental delay, learning disability, autism spectrum disorder
Fragile-XE syndrome	*FMR2/AFF2*	CCG	6–25	>200	5'UTR	X-linked	Learning difficulty
Unverricht-Lundborg disease	*CSTB*	CCCCGCCCCGCG	2–3	>30	5'UTR	AR	Progressive myoclonic epilepsy type 1, Ataxia develops later
Neuronal intranuclear inclusion disease	*NOTCH2NLC*	GGC	<55	>55	5'UTR	AD	Clinical heterogeneous, cognitive impairment, movement disorder, prevalent in East Asians
X-linked dystonia parkinsonism	*TAF1*	CCCTCT	NA	30–55	Intron	X-linked	Dystonia, parkinsonism, founder effect within Filipino population
*C9orf72*-associated ALS/FTD	*C9orf2*	GGGGCC	<30	>500	Intron	AD	Pure frontotemporal dementia, pure amyotrophic lateral sclerosis or combination of the two
Huntington’s disease	*HTT*	CAG	6–34	36–180	Exon	AD	Progressive motor, cognitive, psychiatric disturbance
Huntington disease-like 2	*JPH3*	CAG/CTG	<50	>50	Exon	AD	Progressive movement disorder (including chorea, rigidity) with cognitive impairment. Common in African populations
Familial adult myoclonic epilepsy 1	*SAMD12*	TTTCA	NA (as insertion)	>149	Intron	AD	Adult-onset cortical myoclonus, with seizures in up to a half of patients
Familial adult myoclonic epilepsy 2	*STARD7*	ATTTC	NA (as insertion)	>274	Intron	AD	Finger, hand tremor with later-onset myoclonus and generalised tonic-clonic seizures
Familial adult myoclonic epilepsy 3	*MARCHF6*	TTTCA	NA (as insertion)	>600	Intron	AD	Adult-onset cortical tremor with epilepsy
Familial adult myoclonic epilepsy 4	*YEATS2*	TTTTA/ TTCA	NA (as insertion)	>829 or >221	Intron	AD	Adult-onset cortical tremor with epilepsy
Familial adult myoclonic epilepsy 6	*TNRC6A*	TTTCA	NA (as insertion)	Insertion	Intron	AD	Adult-onset myoclonic epilepsy
Familial adult myoclonic epilepsy 7	*RAPFEG2*	TTTCA	NA (as insertion)	Insertion	Intron	AD	Adult-onset myoclonic epilepsy
Myotonic dystrophy type 1	*DMPK*	CTG	5–37	>50 to ~2000	3'UTR	AD	Mild: cataracts, mild myotonia. Classic: weakness, myotonia, cataracts, cardiac abnormalities. Congenital: hypotonia, severe weakness at birth, respiratory difficulties.
Myotonic dystrophy type 2	*CNBP/ZNF9*	CCTG	<27	>75 to ~11 000	Intron	AD	Myotonia, weakness, cardiac conduction abnormalities, cardiomyopathy, insulin resistance, cataracts, hypogammaglobulinaemia
Oculopharyngeal muscular dystrophy	*PABPN1*	GCG	<10	>12–17	Exon	AD	Ptosis and dysphagia
Oculopharyngeal myopathy with leukoencephalopathy	*NUTM2B-AS1*	CGG	3–16	40–60	5'UTR	AD	Ptosis, ophthalmoplegia, dysphagia, dysarthria
Oculopharyngodistal myopathy 1	*LRP12*	CGG	13–45	90–130	5'UTR	AD	Adult-onset ptosis, ophthalmoplegia, facial, distal limb weakness, dysphagia
Oculopharyngodistal myopathy 2	*GIPC1*	CGG	12–32	97–120	5'UTR	AD	Slowly progressive distal weakness, ophthalmoplegia, facial and bulbar weakness
Kennedy’s disease (spinal and bulbar muscular atrophy)	*AR*	CAG	11–24	40–62	Exon	X-linked	Lower motor neurone and bulbar weakness. Androgen insensitivity: gynaecomastia, testicular atrophy

For mode of inheritance, AD, autosomal dominant and AR, autosomal recessive. ALS, amyotrophic lateral sclerosis; FTD, frontotemporal dementia. Phenotype and genotype descriptions, normal range and pathogenic range repeat sizes derived from Online Mendelian Inheritance in Man, GeneReviews and Bennett *et al*.^39^ Some of the repeat expansion disorders are insertional repeat expansions where the pathogenic repeat motif is not present in the reference genome or in unaffected individuals.

The size of the repeat motif is also important, with trinucleotide repeat expansions being the most common, followed by hexanucleotide repeats. Tetranucleotide (myotonic dystrophy type 2) and pentanucleotide (SCA10, SCA31) repeat expansions also exist. The frequency of repeat expansions of particular motif size reflects the propensity of an STR to expand such that disease-causing trinucleotides have mutation rates 4–7 times greater than tetranucleotides.^41^ Likewise, it is important to characterise the inheritance mode and whether a repeat expansion has to be present on both or one allele to cause disease.

While the overall length of the repeat expansion is important in disease, it is helpful to recognise whether the repeat is uninterrupted and ‘pure’, or whether there are interruptions to the sequence.^42 43^ For example, in SCA2, individuals who have more CAA interruptions within the CAG repeat sequence are more likely to present with an extrapyramidal rather than an ataxia phenotype.^44 45^ Lastly, the size of the repeat can change when transmitted to the next generation in a process of meiotic instability, or across different cell types through somatic instability.^17 43^ The number of interruptions is one of the factors determining the extent of this instability with implications for pathogenesis.^17 42^ These molecular features should be considered in the understanding of repeat expansion disorders.

## Clinical features of repeat expansion disorders

Despite variability in the location, size and repeat motif across different repeat expansions, these disorders share common characteristics that are important to recognise in a clinical setting to aid diagnostic testing and management.

### Clinical heterogeneity of disease proportional to repeat size

Variations in repeat size underlie the clinical heterogeneity of disease within the same pedigree. Repeat expansion disorders should be suspected in families in which individuals have a spectrum of clinical manifestations. For example, congenital myotonic dystrophy is a more severe neuromuscular disorder with multisystemic involvement compared with adult-onset myotonic dystrophy.^46^ Within a family, while larger repeats tend to be associated with earlier onset and more severe disease, penetrance, the likelihood of a clinical condition occurring as a result of a particular genotype, may also differ with repeat size and contributes to clinical heterogeneity. Furthermore, given other factors associated with the pathogenicity of a repeat expansion including the repeat structure and presence of interruptions as discussed, it is difficult to counsel individuals clinically based on the repeat size alone.

### Clinical heterogeneity at intermediate range of repeat size

**Figure 2:**
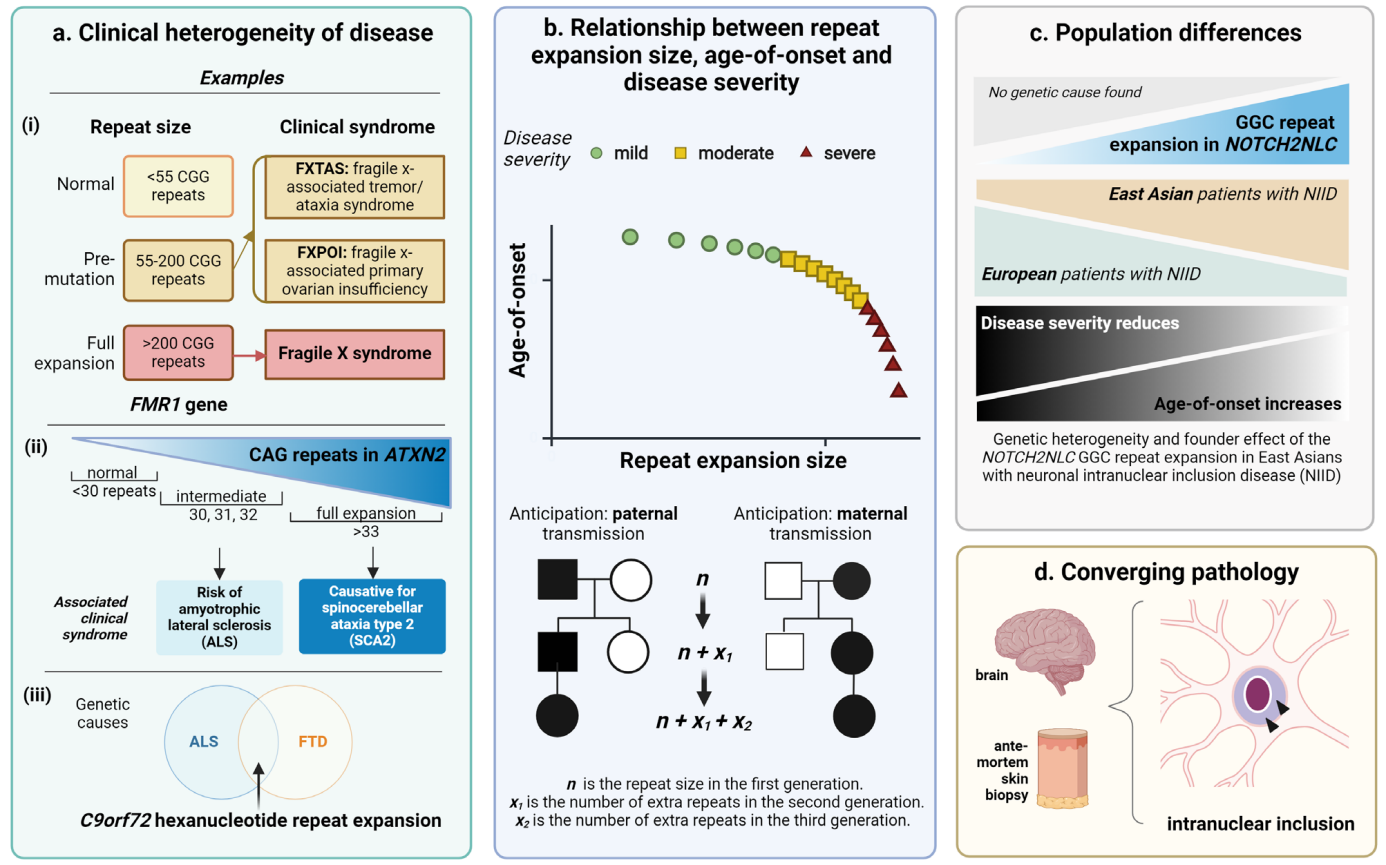
General features of repeat expansion disorders. (**a**) Clinical heterogeneity is often present within the same repeat expansion: (i) Within *FMR1*, different clinical entities are associated with different repeat sizes. (ii) Within *ATXN2*, an intermediate range repeat expansion is associated with increased risk of ALS and a full range expansion causes spinocerebellar ataxia type 2. Therefore, accurate estimation of the repeat size is important for diagnosis. (iii) The *C9orf72* hexanucleotide repeat expansion is a common genetic cause of both amyotrophic lateral sclerosis (ALS) and frontotemporal dementia (FTD) independent of the size of the repeat expansion. (**b**) Schematic showing the inverse relationship between age of disease onset and size of the repeat expansion. The severity of disease typically increases with increasing size of the repeat expansion. This is due to meiotic instability either in the paternal or maternal germline for that particular repeat, whereby n is the overall repeat size in the first generation and x is the number of repeats in addition to the n repeat size in each successive generation, in line with repeat instability associated with anticipation of the clinical presentation. (**c**) A population effect of repeat expansion disorders highlights genetic heterogeneity within one disease entity. This is exemplified in neuronal intranuclear inclusion disease (NIID). NIID is a clinical heterogeneous neurodegenerative condition characterised by eosinophilic neuronal intranuclear inclusions in both neuronal and non-neuronal cells. The *NOTCH2NLC* GGC repeat expansion is a common cause of NIID in individuals of East Asian ancestry who tend to have later-onset, milder disease presenting with cognitive decline or a neuromuscular phenotype. In Europeans, this repeat expansion is rare and disease is more severe, multisystemic and earlier in onset. (**d**). Convergence of different repeat expansion disorders on a common pathological feature. For example, ubiquitinated eosinophilic intranuclear inclusions are seen in brain tissue (and sometimes in antemortem skin biopsy) for a variety of different disorders including NIID and fragile X-associated tremor/ataxia syndrome (FXTAS). Created with Biorender.com.

Distinct phenotypes dependent on repeat size are exemplified in *FMR1-*related disorders (figure 2a.i). In a fully expanded repeat of >200 CGG repeat units, the syndrome is fragile X syndrome, where affected males present with developmental delay and intellectual disability.^47^ In the premutation range of 55–200 CGG repeats, a distinct clinical syndrome of fragile X-associated tremor/ataxia syndrome (FXTAS) characterised by late-onset ataxia, intention tremor with or without parkinsonism and cognitive impairment occurs mainly in men, but also among a fifth of females who are heterozygous for the premutation range expansion.^47^ Furthermore, a distinct entity of fragile X-associated primary ovarian insufficiency before age 40 is also described in a fifth of females who carry one premutation range repeat.^47^ Another entity of fragile X-associated neuropsychiatric disorder has also been characterised in a proportion of individuals who are premutation carriers.^47^ Likewise, within *ATXN2*, an intermediate range repeat expansion is associated with increased risk of ALS and a full range expansion with SCA2 (figure 2a.ii).^48^ Therefore, an accurate estimate of the repeat size is important for genetic diagnosis.

### Clinical heterogeneity irrespective of repeat size

While the variable phenotypes predominantly reflect differing repeat sizes, different phenotypes may be associated with the same expansion irrespective of repeat size. This is demonstrated by the *C9orf72* hexanucleotide repeat expansion associated with either FTD or ALS, or both^19 20^ (figure 2a.iii).

### Anticipation

Given instability of the repeat during meiosis, the expansion size may enlarge in each subsequent generation, associated with clinical anticipation, where there is an earlier onset and more severe phenotype within affected offspring compared with their parents (figure 2b). This is a particular phenomenon of dominant repeat expansion disorders and exemplified in Huntington’s disease, where juvenile onset forms are associated with more than 55 CAG repeats compared with typically fewer than 55 repeats in adult-onset disease.^49^ Anticipation is more noticeable from paternal transmission due to repeat instability in spermatogenesis.^50^ On the other hand, in myotonic dystrophy type 1, a child with early-onset severe disease is more likely to inherit the expanded allele from the mother, although anticipation can occur with both paternal and maternal transmission.^46^ Thus, altered disease onset and severity in the offspring would provide a clue for suspecting a repeat expansion disorder. This also has implications in genetic counselling. Furthermore, it is important to recognise that repeat expansion instability across generations may mean that there appears to be a lack of family history in people presenting with late-onset disease or generation skipping due to intergenerational instability and reduced penetrance. Thus, a lack of family history should not preclude clinical suspicion of a repeat expansion disorder. This is seen in adult-onset repeat expansion disorders such as SCA27B associated with a GAA repeat expansion in *FGF14* where a significant proportion of patients have no family history of disease.^26^

### Population differences

Repeat expansion disorders often exhibit population differences in their prevalence due to a founder effect of a particular risk haplotype containing the repeat expansion in a particular geographical location. Population differences are appreciable in the genetic cause of SCAs such as SCA3 being particularly prevalent in those with Portuguese ancestry, SCA4 in those with Swedish ancestry and SCA31 in those with Japanese ancestry.^51 52^ Other examples that illustrate the founder effect across repeat expansion disorders include the high prevalence of *C9orf72*-assciated disease in European individuals,^53^ X-inked dystonia-parkinsonism in individuals of Filipino ancestry,^54^ Huntington’s disease-like 2 being an common phenocopy of Huntington’s disease in those with African ancestry^55^ and dentatorubral-pallidoluysian atrophy being common in Japanese populations.^56^ Recently, this has been exemplified in neuronal intranuclear inclusion disease (NIID), a clinically heterogeneous neurodegenerative condition characterised by eosinophilic ubiquitinated neuronal intranuclear inclusions in both neuronal and non-neuronal cells. In East Asians, the disease is secondary to a prevalent *NOTCH2NLC* 5’UTR GGC repeat expansion, associated with later onset disease (figure 2c).^57 58^ In Europeans, disease is earlier in onset, more severe and with a mostly undetermined genetic cause.^59^ On a population level, repeat expansion in *NOTCH2NLC* is ultra rare in Europeans.^59^ Thus, it is important to be mindful of population-specific differences when considering repeat expansion disorders and the genetic heterogeneity associated with a common phenotypic entity.

### Converging pathology

Different repeat expansion disorders may converge on a common pathological endpoint irrespective of the clinical presentation or underlying genetic diagnosis (figure 2d). For example, neuronal intranuclear inclusions that are eosinophilic and stain positive for p62 and ubiquitin are present in postmortem examination of FXTAS and NIID brains,^57 58 60^ and more latterly, in SCA4 brains.^34 35^ These diseases are secondary to GGC repeat expansions, although in different genes, and in both non-coding and exonic regions. In *NOTCH2NLC* repeat expansion-positive NIID, these inclusions have also recently been identified on skin biopsy enabling antemortem diagnosis.^61^ Thus, it is worth considering the presence of an underlying repeat expansion disorder where there is additional evidence for the presence of intranuclear inclusions which may represent repeat-containing RNA foci trapped in the nucleus or translated polypeptide.

## Update on recently discovered repeat expansion disorders

Table 1 presents a non-exhaustive list of repeat expansion disorders associated with neurological disease and their corresponding clinical and molecular features. Figure 1c^62 63^ graphically summarises this. Over the last 5 years, there has been a swift rise in the number of repeat expansions discovered, driven both by bioinformatic tools and the availability of long-read sequencing techniques.^19^ Here, we briefly outline a few recent important discoveries of relevance in neurology.

While CANVAS has now become a well-known clinical entity, in which testing for the causative biallelic pentanucleotide repeat expansion in *RFC1* is now available in the NHS, highlighting the rapid translation of research findings, further work is underway to streamline a diagnostic test for *FGF14* repeat expansion in SCA27B outside of the research setting. This repeat expansion disorder was characterised at the end of 2022 in which affected individuals present with a slowly progressive predominant gait ataxia in late adulthood with median age of onset at 60 years of age.^26 27^ Up to half of individuals first present with episodic symptoms of ataxia, vertigo, diplopia and oscillopsia.^26^ The causative variant is a heterozygous GAA expansion of more than 300 repeats in the first intron of *FGF14*.^26^ It is worth considering SCA27B in individuals with previously undiagnosed late-onset ataxia (often without family history) as it has been estimated to be responsible for at least 9% of unsolved ataxia cohorts, rising to 60% in individuals of French Canadian ancestry.^26^ Furthermore, preliminary studies have shown some symptomatic benefit in the use of 4-aminopyridine for symptoms of ataxia and downbeat nystagmus.^64^

The last 5 years has also seen a rise in the number of GGC/CCG repeat expansion disorders being described. These include *NOTCH2NLC* repeat expansion in NIID and intronic CCG repeat expansions in *NUTM2B-AS1, LRP12* and *GIPC1* causing oculopharyngeal myopathy with leukoencephalopathy, oculopharyngodistal myopathy (OPDM) types 1 and 2, respectively.^57^ These disorders have substantial overlapping clinical, neuroimaging and pathological features likely stemming from similar pathogenic mechansisms.^65^ Recently, exonic GGC repeat expansion in *ZFHX3* has also been described to be associated with the previously molecularly undiagnosed SCA4.^32^,^35^ These disorders join the ranks of NIID to form a new disease entity of polyglycine disorders.^65 66^ Understanding these disorders collectively is important as they are typically adult-onset, slowly progressive conditions with overlaps in clinical presentation, such as a similar pattern of weakness seen in a large proportion of East Asian individuals with *NOTCH2NLC-*repeat-positive NIID as individuals with OPDM. Moreover, as discussed, there are also many similarities pathologically in terms of the presence of intranuclear inclusions in both neuronal and non-neuronal cells. Thus, the understanding of a common disease entity will aid diagnosis and preparation for therapeutic development in such disorders.

The discovery of novel repeat expansions in the research setting has outstripped the availability of validated diagnostic testing for these disorders in clinical laboratories. Thus, it is worth noting that genetic testing for some of the newer repeat expansions is unavailable within the remit of the healthcare system.

## How are repeat expansions tested in the laboratory?

### Conventional approaches

**Figure 3:**
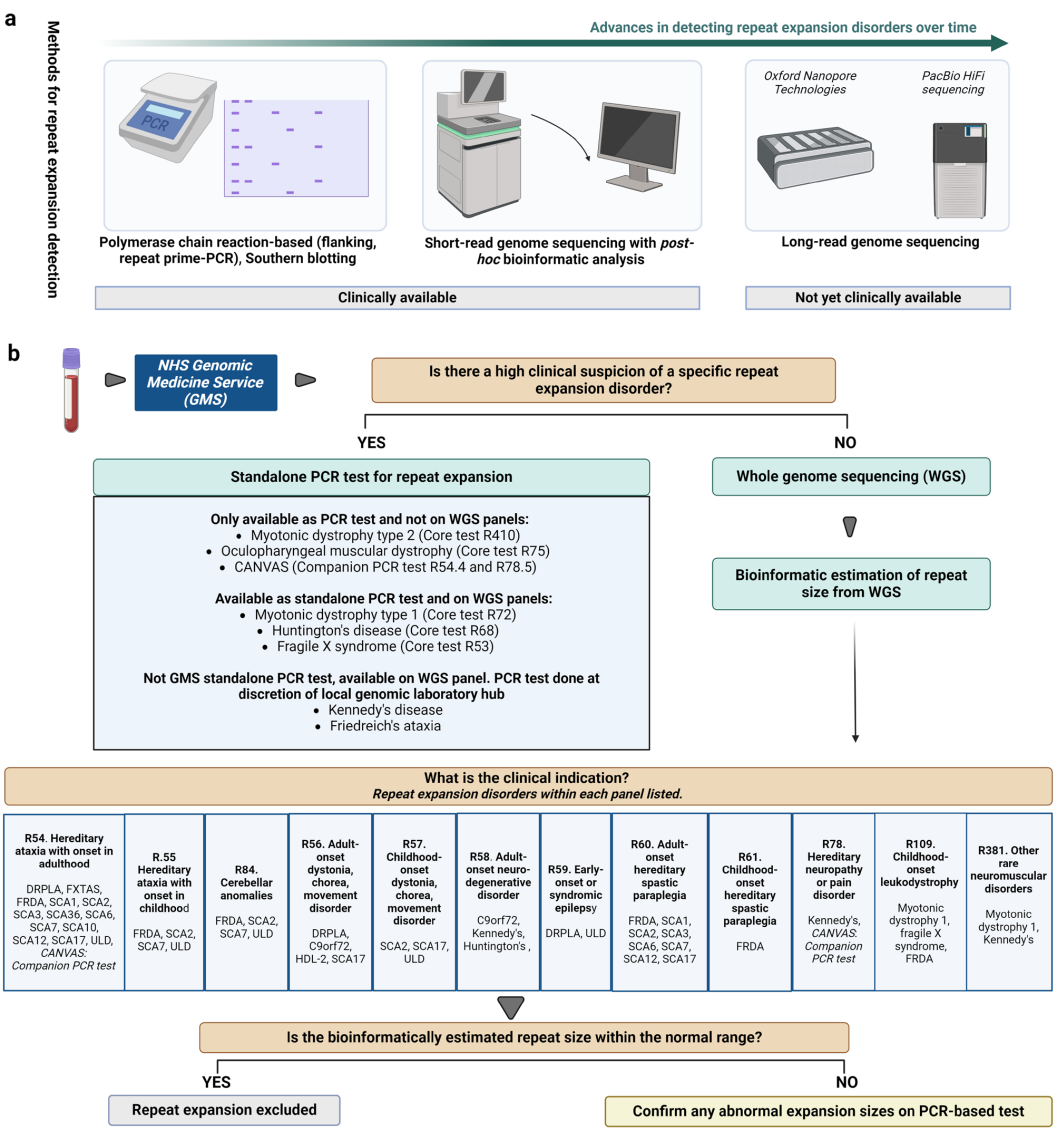
Methods for detection of repeat expansions. (**a**) Evolution of methods used for detection of repeat expansions: from molecular, PCR-based approaches to bioinformatic tools applied to short-read exome or genome sequencing data to recent development of long-read sequencing technologies. (**b**) Clinical pathway for testing for repeat expansions within the National Health Service (NHS) Genomic Medicine Service (GMS). Note that the panel numbers and GMS pathway may be subject to change. The GMS website should be consulted for up-to-date panels. The panels provided here are for illustrative purposes of the principles of repeat expansion detection. DNA extracted from blood is processed for repeat expansion testing. If a particular repeat expansion disorder is suspected, then a standalone PCR with or without Southern blotting is requested if it falls into the remit of one of the listed disorders. If the clinical indication falls within any of 12 listed gene panels, then short-read whole-genome sequencing is carried out. Within each of the panels, integrated estimation of repeat size of a specific disorder within the panels listed by ExpansionHunter is provided. Further molecular confirmation is carried out if the bioinformatically estimated repeat size is above or falls just below the pathogenic threshold for repeat expansion. ALS, amyotrophic lateral sclerosis; CANVAS, cerebellar ataxia, neuropathy, vestibular areflexia syndrome; DRPLA, dentatorubral-pallidoluysian atrophy; FTD, frontotemporal dementia; FRDA, Friedreich’s ataxia; FXTAS, fragile X-associated tremor/ataxia syndrome; HLD-2, Huntington disease-like 2; SCA, spinocerebellar ataxia; ULD, Unverricht-Lundborg disease. Created with BioRender.com.

Conventionally, the detection of repeat expansion disorders in the clinical diagnostics services has relied on low-throughput PCR-based techniques with and without the additional need for Southern blotting to size very large expansions (figure 3a). For example, in an individual presenting with an autosomal dominant ataxia syndrome, the first line of screening would usually be with standardised PCR protocols for SCA 1, 2, 3, 6, 7 and 17. In practice, this involves six different protocols in the laboratory setting to specifically amplify the repeat-containing DNA then visualising the repeat using fragment analysis through capillary electrophoresis, which is both time-consuming and limited in the number of loci assessed. As these disorders are clinically and genetically heterogeneous, the yield for picking up rarer repeat expansion disorders is low and slow given the serial sequential testing required.

### Recent advances

It has been traditionally difficult to detect repeat expansions using next-generation sequencing data as DNA is broken into short fragments or reads ranging between 50 and 300 bases before being sequenced. Thus, repetitive regions longer than the read length cannot be tested accurately. However, a recent study using a bioinformatic tool called ExpansionHunter^67 68^ that estimates repeat sizes at known STR loci using short-read exome or genome sequencing data (figure 3a) was shown to be able to detect expanded and non-expanded alleles at 13 loci of the most prevalent repeat expansion disorders with high specificity (100%) and sensitivity (99.1%) in participants enrolled in the 100 000 Genomes Project.^28^ There was high concordance with repeat size estimates from conventional PCR-based methods although larger repeats such as *FMR1* associated with fragile X syndrome and *DMPK* repeat expansion associated with myotonic dystrophy type 1 were underestimated.^28^ ExpansionHunter and other such bioinformatic tools have also enabled the ability to estimate STR sizes across different populations at scale, shedding light on population differences in STR structure and size.^69^ Other bioinformatic tools also exist to detect STR repeat expansions using short-read WGS data for clinical use and are reviewed in more detail elsewhere.^39^

### Current approach in clinical testing

As such, it is now tractable to estimate STR sizes of certain repeat expansions diagnostically from short-read WGS data. This has been implemented in clinical practice and within the NHS Genomic Medicine Service in England. Scotland (Scottish Strategic Network for Genomic Medicine), Wales (All Wales Medical Genomics Service) and Northern Ireland have individualised ways of genomic testing, details of which are explained in the following link: https://www.genomicseducation.hee.nhs.uk/genotes/knowledge-hub/genomic-testing-in-the-devolved-nations/. Repeat expansion testing by PCR-based methods can also be sent to and run by diagnostics laboratories in England if required, via the genetic service of the respective nation.

First, if a specific repeat expansion disorder is suspected based on the clinical phenotype, then a standalone PCR-based test is requested if it falls within the remit of one of the listed disorders that are common enough to exist as a standalone test (figure 3b). Some loci, such as for the repeat expansion associated with myotonic dystrophy type 2 and OPMD only exist as standalone PCR tests as they have not been validated on the WGS bioinformatic analysis. Otherwise, most of these loci are also on one or more WGS panels. For example, the CTG repeat in *DMPK* associated with myotonic dystrophy type 1 is in seven gene panels including congenital myopathy panel (R81) and intellectual disability panel (R29) (https://nhsgms-panelapp.genomicsengland.co.uk/entities/DMPK_CTG), but also as a standalone core PCR test (R72). Lastly, some repeat expansions such as Kennedy’s disease and Friedreich’s ataxia can only be tested using bioinformatic analysis through gene panels in the Genomic Medicine Service (discussed below) and not as standalone PCR tests. However, standalone PCR tests can often be done at the discretion of the local genomic laboratory hub and varies across hubs. Further discussion with the local laboratory is therefore helpful to ensure that the correct tests are being requested.

If the clinical suspicion is not confined to a particular disorder, repeat expansion screening using ExpansionHunter applied to WGS data falls into wider screening panels directed by the clinical indication as with non-repeat expansion disorders (figure 3b). For example, for an individual presenting with ataxia in adulthood, the ‘hereditary ataxia with onset in adulthood’ gene panel (R54) will be selected and genes in which known pathogenic variants are associated with disease are assessed using WGS data. This panel will include screening for repeat expansions associated with SCA1, 2, 3, 6, 7, 10, 12, 17, Friedreich’s ataxia, dentatorubral-pallidoluysian atrophy, Unverricht-Lundborg disease and FXTAS (figure 3b). The estimation of STR sizes associated with these disorders is implemented through the bioinformatic tool ExpansionHunter applied to WGS data. If ExpansionHunter detects a repeat in the pathogenic range, PCR-based tests are used to confirm the molecular diagnosis. Therefore, it is important to check whether the desired repeat expansions screened are covered in the corresponding gene panels. This method enables multiple repeat loci to be interrogated simultaneously using one genetic test (WGS) rather than sequential single repeat expansion testing using conventional molecular approaches. Furthermore, WGS covers both coding and non-coding repeat expansions.

A caveat is that detection of several repeat expansion disorders is not validated using WGS data. Therefore, they need to be noted as additional tests on the request. This includes the *RFC1* expansion in CANVAS. While this is not available on the WGS panels, it can be requested for any patient having the R54 (adult-onset ataxia) or R78 (hereditary neuropathy) panels as a companion test by PCR (R54.4 and R78.5). Similarly, as discussed, testing for OPMD (*PABPN1*, R75) and myotonic dystrophy type 2 (*CNBP (ZNF9),* R410) are only available through PCR-based tests and need to be requested additionally to the panels of interest.

It is worth noting that while the above description of panels is valid at the time of writing and helpful to illustrate the approach to genetic testing, gene panels are constantly updated by the Genomic Medicine Service. To ensure adequate testing, it is advisable to refer to the most up-to-date panel sign-offs on https://nhsgms-panelapp.genomicsengland.co.uk/ and consult the local genomic laboratory hub to account for regional variability, if any (table 2 shows some useful resources).

**Table 2:** Useful resources

URL	Description
https://nhsgms-panelapp.genomicsengland.co.uk/	NHS Genomic Medicine Service (GMS) Signed Off Panels Resource. Using the ‘STR’ entity, a list of STRs screened for, their associated phenotypes and gene panel is listed with further information.
https://www.england.nhs.uk/publication/national-genomic-test-directories/	National Genomic Test Directory with gene panels
https://www.genomicseducation.hee.nhs.uk/genotes/	Information from the National Genomics Education Programme
https://www.ncbi.nlm.nih.gov/books/NBK1116/	GeneReviews: point-of-care resource for genetic conditions
https://www.omim.org/	Online catalogue of human genes and genetic disorders

### Future advances

For the insertional repeat expansions where the STR is not present in the reference genome, ExpansionHunter Denovo^70^ estimates sizes of repeats without prior information of STR location and has been used to detect several repeat expansions recently including SCA27B.^26^ However, testing for novel repeat expansion without *a priori* knowledge of the STR is not currently available in clinical practice. Lastly, the new era of long-read sequencing technologies through the Oxford Nanopore platforms and PacBio Single Molecule Real-Time Sequencing technologies (figure 3a) has driven the successful discovery of several novel repeat expansion disorders in recent years. This technology uses longer reads of tens to thousands of kilobases to span complex regions without needing to fragment DNA, enabling more accurate sequencing of repetitive genomic regions. Although these powerful tools are not currently used in clinical practice, its development for incorporation in diagnosis is currently being assessed. Moreover, long-read sequencing has crucially allowed the human genome to be sequenced to completion^8^ and generate a better map of complex variants on a population level to aid variant interpretation.^71^

Box 2 provides case examples illustrating the clinical features of repeat expansion disorders and the pathway to molecular diagnosis.

Box 2 **Case examples**

**Case 1.**

A 10-year-old girl was investigated for intellectual disability and a complex multisystem neurological disease. Both her parents were asymptomatic. ExpansionHunter applied to WGS data revealed a very large repeat expansion of 99 CAG repeats in *ATXN2* confirming a diagnosis of early-onset SCA2, that had a different presentation to adult-onset SCA2.

**Case 2.**

A child under the age of 5 presented with ataxia and global developmental delay was found to have a large repeat expansion of 120 CAG repeats in *ATXN7* consistent with early-onset SCA7 after bioinformatic analysis of multiple loci using ExpansionHunter applied to WGS data. Of note, the child’s parents had been well at the time WGS was completed for the proband but 2 years after this, her father presented with ataxia and was subsequently also found to have a heterozygous CAG repeat expansion in *ATXN7*.

**Case 3.**

A previously well man in his 60s had presented to the neurology clinic with tremor. On examination, he had bilateral intention tremor. Family history revealed that his grandson was being investigated for learning difficulties and behavioural issues. An MRI brain scan showed bilateral symmetric T2 hyperintensities in the middle cerebellar peduncles. Clinical suspicion for FXTAS was high. DNA was directly analysed for the *FMR1* CGG repeat expansion using repeat-prime PCR (indication under Fragile X syndrome) and a premutation of 110 CGG repeat units was confirmed. Subsequently, his grandson was diagnosed with fragile X syndrome after detection of a full-range repeat expansion in *FMR1*.

These cases highlight intergenerational instability and anticipation of the repeat expansion meaning that often there may be no family history and ExpansionHunter is a reliable screening tool for multiple loci in unexplained cases. Furthermore, the cases demonstrate the clinical heterogeneity within the same family.

## Conclusions

The last decade has seen remarkable improvements in bioinformatic and sequencing techniques that have culminated in the use of whole-genome sequencing in routine clinical care. The next age of neurogenetic diagnosis will focus on unsolved cohorts and ultimately, targeted therapeutic development, driven by common pathogenic mechanisms at the DNA level. These apparently intractable cases are, in part, due to complex forms of genetic variation located in previously unexplored and presumed ‘non-coding’ genomic regions. This is evident in the exponential rise in the number of characterised repeat expansion disorders within apparently non-coding regions of the genome over the last 5 years. Given that repeat expansions underlie a significant proportion of neurological conditions including monogenic forms of neurodegenerative disorders, neurologists are at the forefront of advancing our understanding of this collective group of diseases.

**Key points**

- Most repeat expansion disorders manifest as neurological disease.
- Repeat expansion disorders have shared features of anticipation, clinical heterogeneity (both dependent and independent of the repeat size) and founding population differences, which provide clues to their diagnosis.
- There is sometimes no associated family history due to intergenerational repeat size instability.
- Laboratory testing for particular disorders was conventionally performed through PCR-based methods but can now be performed through analysis of whole-genome sequencing data.

**Further reading**

- Morris HR, Houlden H, Polke J. Whole-genome sequencing. *Pract Neurol* 2021;21(4):322–7. doi: 10.1136/practneurol-2020-002561.
  - Practical guide on using whole-genome sequencing in clinical practice.
- Smedley D, Smith KR, Martin A, et al. 100 000 Genomes Pilot on Rare-Disease Diagnosis in Health Care - Preliminary Report. *N Engl J Med* 2021;385(20):1868–80. doi: 10.1056/NEJMoa2035790.
  - Diagnosis of rare genetic disorders from the NHS England 100 000 Genomes Project.
- Ibañez K, Polke J, Hagelstrom RT, et al. Whole genome sequencing for the diagnosis of neurological repeat expansion disorders in the UK: a retrospective diagnostic accuracy and prospective clinical validation study. *Lancet Neurol* 2022;21(3):234–45. doi: 10.1016/s1474-4422(21)00462-2.
  - Validity of using bioinformatics tools to estimate repeat expansion size from whole-genome sequencing data that led to a change in detection of repeat expansion disorders within the NHS England Genomic Medicine Service.

## Data Availability

No data are available.
